# Beyond skills: Reimagining clinical education for a transformative future

**DOI:** 10.4102/sajp.v82i2.2377

**Published:** 2026-06-26

**Authors:** Hellen Myezwa, Tania R. van der Merwe, Tonderai W. Shumba

**Affiliations:** 1 Department of Physiotherapy, School of Therapeutic Sciences, Faculty of Health Sciences, University of the Witwatersrand, Johannesburg, South Africa; 2 Higher Education Studies, Centre for Learning, Teaching and Development, University of the Witwatersrand, Johannesburg, South Africa; 3 Department of Occupational Therapy and Physiotherapy, School of Allied Health Sciences, Faculty of Health Sciences and Veterinary Medicine, University of Namibia, Windhoek, Namibia

Clinical education in the health professions stands at a critical juncture. Within a Global South context, the unabating Pareto principle of 80% of the population relying on resource-scarce public health, vis-à-vis roughly 20% of the population having access to private healthcare (Abdullah et al. 2024), global technological innovation and providing epistemically just clinical education compel educators to rethink how future professionals are prepared for practice. This special edition brings together diverse scholarly contributions that collectively reimagine clinical education for a transformative future.

Across the articles included in this edition, three interconnected levels of engagement with clinical education emerge – the first concerns learning design. Contributions explore the expanding role of pre-clinical simulation, digital innovation and technology-enhanced learning within therapeutic health sciences curricula. The second level of contributions focuses on capability formation and professional identity development. Here, authors interrogate pedagogical approaches that move beyond procedural competence towards integrated, reflective practice. Emphasis is placed on the intentional incorporation of theoretical frameworks, such as the International Classification of Functioning, Disability and Health (ICF), into curricula, as well as on cultivating students’ capacity for accurate self-reflection and reflexivity. Clinical education, in this sense, is not merely about performing tasks competently; it is about shaping practitioners who can think critically, act ethically and adapt responsibly in complex contexts. The third level engages system accountability and curriculum responsiveness. Contributions examine how policy frameworks inform curriculum design, the importance of embedding preventative and public health competencies and the need for greater coherence in standards for clinical competency assessment and external examination. Together, these discussions highlight that clinical education does not operate in isolation; it is embedded within broader regulatory, institutional and societal systems that demand accountability and alignment with population health priorities.

Collectively, these articles prompt a reconsideration of what clinical education fundamentally entails. While clinical education is often described simply as students ‘*practising what they have learnt in the real world*’, the scholarship in this edition demonstrates that it extends far beyond skills transfer. Clinical education is the deliberate cultivation of competent, compassionate professionals capable of navigating uncertainty and delivering patient-centred care. This aligns with global competency frameworks, including the World Health Organization’s competency guidance (WHO 2022) and the Canadian Medical Education Directives for Specialists (CanMEDS) framework (Royal College of Physicians and Surgeons of Canada 2026), which articulate roles such as communicator, collaborator, leader, health advocate, scholar and professional. Meaningful learning across these domains depends on intentional scaffolding; within individual teaching moments, yes, but also across the entire arc of a learner’s development.

Several contributions also foreground the interplay between pedagogy, technology and context in shaping inclusive and democratic learning environments. For Generation Z learners, whose world is fast-moving and unpredictable, cocreation and collaboration are no longer optional. They are essential conditions for responsive engagement in the workplace and interactive relevance in the learning environment (e.g. Licas & Torres 2024; Omland et al. 2025). Educational design must therefore be responsive, flexible and dialogical.

Assessment within clinical education is another critical theme. Assessment should not be seen as a mechanism for judgement, but as a catalyst for competence. Assessment needs to be reframed as feedback-rich, diagnostic and a developmental tool (Dodson & Thompson-Hairston 2025). While change and disruption often provoke discomfort, they also compel us to question entrenched practices and embrace the agency we hold as educators to do better.

Importantly, clinical education is not about possessing all the answers. It is about creating the conditions for students’ ‘lightbulb’ moments. Through teamwork, transdisciplinary exploration and a willingness to reflect, fail and try again, we begin to see ourselves not just as instructors, but as facilitators of transformative learning experiences through praxis and enacting practical wisdom (Aristotle 1998).

Within this discourse, real challenges continue to be confronted: strained resources, growing workloads and the well-being of staff juggling teaching, clinical workload and supervision. One pivotal question is: Should we continue trying to do more with less, or should we have the courage to do *different* with less? Such a transformative future may be prioritising depth over breadth, coherence over fragmentation and collaboration over isolation.

Ultimately, a transformative future in clinical education is not confined to curriculum design. It is about supporting students through their transitions, helping them make meaning of their learning and nurturing a genuine sense of belonging. Because belonging strongly predicts success (Zaky 2024; Zariff & Stallman 2025), it requires us to unpack the hidden curriculum and the assumptions we may carry or uncritically reproduce without realising it. While clinical reasoning is essential, inconsistent clinical teaching methods illuminate the need for consensus in structured, evidence-based approaches to enhance clinical education. Strengthening clinical reasoning is critical to prepare physiotherapy graduates to be responsive to modern health system demands (Sharma et al. 2025).

We hope this special edition on clinical education can help pave the way for more meaningful engagements and spark further debates on improving physiotherapy clinical education. In the end, clinical education should be a space where students feel heard, supported and empowered to become not only capable professionals but active agents of change. Our responsibility as educators is clear: to cultivate learning that transforms, inspires and sustains both our students and ourselves.
